# Synthesis, Molecular Docking and Cytotoxic Activity Evaluation of Organometallic Thiolated Gold(I) Complexes

**DOI:** 10.22037/ijpr.2020.1101118

**Published:** 2020

**Authors:** Zeinab Faghih, Alireza Yazdani Kachoei, Hossein Alizadeh, Suphia Emamdoost, Shima Shirkhan, Masood Fereidoonnezhad

**Affiliations:** a *Pharmaceutical Sciences Research Center, Shiraz University of Medical Sciences, Shiraz, Iran. *; b *Toxicology Research Center, Ahvaz Jundishapur University of Medical Sciences, Ahvaz, Iran.*; c *Student Research Committee, Ahvaz Jundishapur University of Medical Sciences, Ahvaz, Iran. *; d *Department of Medicinal Chemistry, School of Pharmacy Ahvaz Jundishapur University of Medical Sciences, Ahvaz, Iran.*

**Keywords:** Synthesis, Gold(I) complexes, Thiolate ligands, Molecular docking, Cytotoxic activity

## Abstract

The complex [(PhCH_2_NC)AuCl], **1**, was prepared by the reaction of [(Me_2_S)AuCl], **A**, with an equimolar amount of benzyl isocyanide (PhCH_2_NC) ligand. Through a salt metathesis reaction, the chloride ligand in **1** was replaced by potassium benzothiazole-2-thiolate (Kbt) and potassium benzoimidazole-2-thiolate (Kbi) to afford complexes (PhCH_2_NC)Au(κ^1^-S-bt)], **2a** and (PhCH_2_NC)Au(κ^1^-S-bi)], **2b**, respectively, which were characterized by NMR spectroscopy. The cytotoxic activities of **2a** and **2b** were evaluated against three human cancer cell lines, including A549 (lung), SKOV3 (ovary), and MCF-7 (breast). Our results indicated that **2a** exhibited comparable cytotoxicity on investigated cell lines with cisplatin. It showed a good anti-proliferative activity with IC_50_ of 19.46, 11.76 and 13.27 μM against A549, SKOV3 and MCF-7 cell lines, respectively. The effects of these complexes on the proliferation of the non-tumorigenic epithelial breast cell line (MCF-10A) showed their good selectivity between the tumorigenic and non-tumorigenic cell lines. Molecular docking simulation studies were also conducted to determine the specific binding site and binding mode of the synthesized gold complexes to DNA and thioredoxinreductase (TrxR) as their proposed targets.

## Introduction

The commercially available platinum-based drugs such as cisplatin, carboplatin, and oxaliplatin suffer from severe side effects and resistance in a wide range of cancers (1, 2). Recently, gold-based complexes, such as auronofin, an anti-rheumatic drug, have received great attentions in the treatment of cancers due to their potent anti-proliferative activity against wide variety of cancer cell lines with different mechanisms of action compared to Pt-based drugs (3).

Two significant oxidation states of gold is +1 and +3 (4). Au(I) as the most stable oxidation state of gold forms usual linear complexes by coordination of two ligands (5). The nature of the coordinating ligands (donor atoms) has an important effect in the stability of the gold(I) complexes (6-9). These complexes usually contain soft donor atoms such as sulfur (10-13), carbon (14-20), and phosphorus (10, 21-23).

Thiol group (RSH) and their corresponding anion forms, called thiolate (RS-), as a chemically unique ligands, can be used as a source of sulfur donor ligands in a variety of organometallic complexes (24-27). To date, different thiolate gold(I) complexes especially the type of L-Au^I^-SR have been synthesized, so that ligand(L) could be different neutral ligands such as isocyanides (28-31), phosphine (13, 29, 32 and 33), and N-heterocyclic carbenes (NHCs) (34-36). Generally, the presence of thiolate ligands and various L donor ligands in their structure greatly affects their biological activity (13, 32, 34 and 36).

Recent studies demonstrated that the thioredoxin reductase (TrxR) which catalyzed the NADPH-dependent reduction of oxidized thioredoxins, is an effective target for the development of novel antitumor agents (37, 38). Several cytotoxic gold compounds, both gold(I) or gold(III), are potent TrxR inhibitors (39). These enzymes were introduced as the main targets of anticancer gold agents (37). The interaction of the gold complexes with DNA was also proposed for their anticancer properties (40, 41).

Here, in this study, the synthesis, structural characterization, and cytotoxic activity of two new Au(I) complexes with benzyl isocyanide and thiolate ligands (benzothiazole2-thiolate (bt) and benzoimidazole-2-thiolate (bi)) were demonstrated. The cytotoxic activity was evaluated on three different cancer cell lines including human lung (A549), ovarian (SKOV3), and breast (MCF-7) cancer cell lines. Molecular docking simulation studies were also conducted to determine the specific binding site and binding mode of the synthesized gold complexes to DNA and thioredoxinreductase (TrxR) as their proposed targets.

## Experimental

*Materials and methods*

*General procedures and materials*

All reactions were carried out under a nitrogen atmosphere using standard Schlenk techniques. NMR spectra (^1^H and ^13^C{^1^H}) were recorded on a Bruker Avance DPX 400 MHz instrument and referenced to the residual peak of the solvent, *i.e.* CDCl_3_ (^1^H and ^13^C). The chemical shifts (δ) were reported as ppm and coupling constants (*J*) were expressed in Hz. The melting point values were measured by a Buchi 510. The microanalyses were performed using a vario EL CHNS elemental analyzer. Benzyl isocyanide (PhCH_2_NC), benzothiazole-2-thiol (Hbt), and benzoimidazole-2-thiol (Hbi) as well as all the solvents were purchased from Aldrich and used without further purification. Complex [(Me_2_S)AuCl], **A**, was prepared according to literature method (40).

*Synthesis of potassium benzothiazole-2-thiolate (Kbt)*

A solution of benzothiazole-2-thiol (Hbt, 373 mg, 2.23 mmol) in MeOH (10 mL) was added to a solution of KOH (125 mg, 2.23 mmol) in MeOH (5 mL). The resulting yellow solution was stirred at room temperature for 1 h, and then the solvent was completely evaporated. The residue was treated with ^i^PrOH (2 mL) and the resulting yellow solid was filtered and dried. This procedure was also used for preparation of potassium benzoimidazole-2-thiolate (Kbi).

*Synthesis of [(PhCH*_2_ *NC)AuCl], 1*

To a solution of [(Me_2_S)AuCl], **A** (200 mg, 0.68 mmol) in CH_2_Cl_2_ (20 mL), 1 equivalent of PhCH_2_NC (83 μL, 0.68 mmol) was added. The mixture was stirred at room temperature for 1 h and then concentrated (~1 mL) under vacuum, and *n*-pentane (5 mL) was added to give **1** as a white solid, which was filtered and washed with *n*-pentane (2 × 3 mL) and dried. Yield: 197 mg, 83%; m.p. = 138 *°*C. Elem. Anal. Calcd. for C_8_H_7_AuClN (349.57): C, 27.49; H, 2.02; N, 4.01. Found: C, 27.61; H, 2.05; N, 4.06. IR (KBr, cm^-1^): 2260 (s, υ_C≡N_). NMR data in CDCl_3_: δ (^1^H) 4.87 (s, 2H, H^e^), 7.35 (dd, ^3^*J*_HH_ = 7.7 Hz, ^4^*J*_HH_ = 1.4 Hz, 2H, H^a^), 7.46-7.43 (m, 3H, H^b^ and H^c^); δ (^13^C{H}) 48.2 (t, ^1^*J*_CN_ = 7 Hz, C^e^), 127.5 (s, 2C, C^a^), 129.4 (s, C^d^), 129.6 (s, 2C, C^b^), 129.7 (s, C^c^), 135.6 (t, ^1^*J*_CN_ = 26 Hz, C^f^).

*Synthesis of* *(PhCH*_2_*NC)Au(κ*^1^*-S-bt)], 2a *

An equimolar amount of Kbt (59.5 mg, 0.29 mmol) was dissolved in mixture of MeOH/acetone (2/8 mL) and added to a solution of **1** (100 mg, 0.29 mmol) in CH_2_Cl_2_ (15 mL). The reaction mixture was stirred at room temperature for 15 h. Then, the solvent was removed under reduced pressure and the residue was extracted with CH_2_Cl_2_ (10 mL). The obtained colorless solution was filtered through celite and the filtrate was concentrated (~1 mL) under vacuum, and *n*-pentane (5 mL) was added to give **2** as a white solid, which was filtered and washed with *n*-pentane (3 × 3 mL) and dried. Yield: 87 mg, 74%; m.p. = 172 *°*C. Elem. Anal. Calcd. for C_15_H_11_AuN_2_S_2_ (480.35): C, 37.51; H, 2.31; N, 5.83. Found: C, 37.46; H, 2.34; N, 5.85. NMR data in CDCl_3_: δ (^1^H) δ 8.04 – 7.93 (m, 2H), 7.39 (dtd, *J* = 24.0, 7.5, 1.5 Hz, 2H), 7.27 – 7.16 (m, 1H), 7.08 – 6.94 (m, 4H), 4.15 (t, *J* = 0.9 Hz, 2H). δ (^13^C{H}) 168.9, 152.0, 143.0, 139.0, 134.6, 128.5, 128.2, 126.2, 124.5, 121.5, 121.1, 41.3.

*Synthesis of (PhCH*_2_ *NC)Au(κ* ^1^ *-S-bi)], 2b*

The synthesis procedure was as the same as **2a**. Yield: 91 mg, 75%; m.p. = 186 *°*C. Elem. Anal. Calcd. for C_15_H_12_AuN_3_S (463.04): C, 38.89; H, 2.61; N, 9.07. Found: C, 38.82; H, 2.67; N, 9.11. NMR data in CDCl_3_: δ (^1^H) δ 7.98 – 7.87 (m, 2H), 7.47 – 7.40 (m, 1H), 7.25 – 7.12 (m, 3H), 7.08 – 6.94 (m, 4H), 4.15 (t, *J* = 0.9 Hz, 2H). NMR data in CDCl_3_: δ (^1^H) 153.4, 143.0, 140.4, 139.0, 137.5, 128.5, 128.2, 126.1, 124.6, 122.2, 117.4, 111.0, 41.3.

*Biological Assay*

*Cell Lines and Cell Culture*

Human cancer cell lines, MCF-7 (breast cancer), SKOV3 (ovarian cancer), and A549 (non-small cell lung cancer) were purchased from National Cell Bank of Iran (NCBI, Pasteur Institute, Tehran, Iran). All the cells were cultured in RPMI 1640 medium (Biosera), supplemented with 10% fetal bovine serum (FBS; Gibco) and 1% pencilin−streptomycin and were incubated at 37 °C in humidified CO_2_ incubator. MCF10A cells (human breast epithelial cell line) were cultured in DMEM/Ham’s F-12 (GIBCO-Invitrogen, Carlsbad, CA) supplemented with 100 ng/mL cholera toxin, 20 ng/mL epidermal growth factor (EGF), 0.01 mg/mL insulin, 500 ng/mL hydrocortisone, and 5% chelex-treated horse serum.

*MTT Assay*

Cytotoxic activities of **2a** and **2b** were evaluated using standard 3-(4,5-dimethylthiazol-yl)-2,5-diphenyl-tetrazolium bromide (MTT) assay according to a known protocol (25, 42 and 43). Briefly, the cells were harvested and plated in 96-well microplates at a density of 1 × 10^4^ cells per well in 100 μL of complete culture medium. After 24 h of incubation, the cells were treated with five different concentrations of the gold complexes, ranging from 1 to 100 μM in triplicate manner. Each compound was dissolved in DMSO. To avoid bystander cytotoxic effect, the final concentration of DMSO was maintained at about 0.1%. Following 48 h of incubation at 37 °C in humidified CO_2_ incubator, the media were completely removed and replaced with 100 μL of new media containing 0.5 mg/mL MTT solution and the plate were incubated for 3 h at room temperature. The media containing MTT were discarded, and 150 μL of DMSO was added to each well to dissolve the formazan crystals. The plates were then incubated for more 30 min at 37 °C in the dark. The absorbance of individual well was read at 492 nm using a microplate ELISA reader. The data were analyzed using Excel 2013 and CurveExpert 1.4 and the 50% inhibitory concentration of each compound was reported as IC_50. _Each experiment was tested three times for each complex. Data are presented as mean ± SD.

*Molecular docking procedure*

The four different 3D crystal structures of DNA (PDB ID: 1BNA, 1LU5, 3CO3 and 198D) and TrxR (PDB ID: 4CBQ) were retrieved from protein data bank (www.rcsb.org/pdb). Co-crystal ligands were excluded from the structures and the PDBs were checked in terms of missing atom types. Subsequently, MGLtools 1.5.6 was applied to convert these corrected PDB files to PDBQT. The structure of each gold(I) complexes was created by HyperChem Professional (Version 8, Hypercube Inc., Gainesville, FL, USA). Each complex was optimized by molecular mechanic methods (MM^+^) using HyperChem 8, followed by energy minimization calculations at Hartree-Fock (HF) level, using Gaussian 09. The output structures were then converted to PDBQT using MGLtools 1.5.6. The ligands, thereafter, were docked in the active site of DNA and TrxR using an *in-house* batch script (DOCKFACE) of AutoDock 4.2, based on Lamarckian genetic algorithm (44-47). 

A grid box of 60 × 74 × 120 and 40 × 40 × 40 points in x, y, and z directions was built and centered on the ligand in the complex with a spacing of 0.375 Å for 1BNA and 4CBQ, respectively. Cartesian coordinate for 1BNA in x, y, and z was 15.81, 21.31, and 9.88, and for 4CBQ was -4.923, -7.115, and -22.251, respectively. Parameters of metal ions such as gold were added to the gpf and dpf files to be used in the docking calculation. Visualization of the docked pose has been performed by means of AutoDock Tools 1.5.6 and PyMOL molecular graphics program (48).

## Results and Discussion

*Synthesis and Characterization of Complexes*

**Scheme 1:**
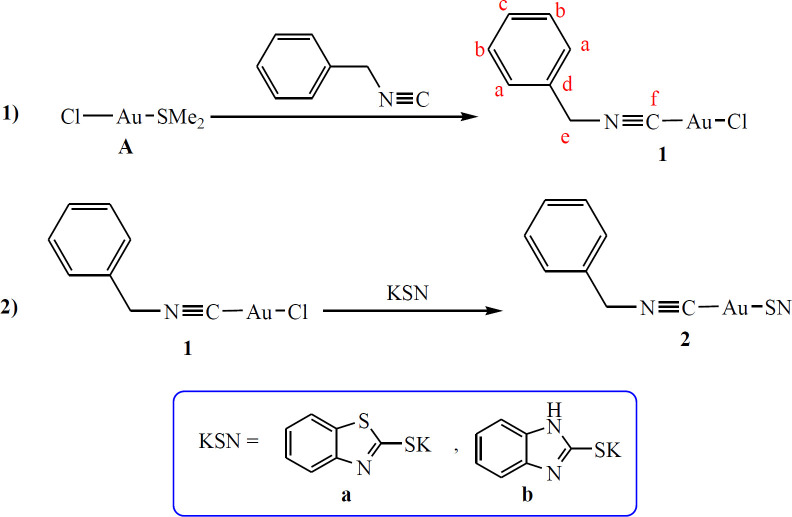
Synthetic route for preparation of **2a** and **2b**

According to the Puddephatt report the precursor complex [(Me_2_S)AuCl], **A**, was prepared (49). The SMe_2_ ligand in **A** is a good leaving group and can be readily substituted by one equivalent of benzyl isocyanide (PhCH_2_NC) and afforded the corresponding complex [(PhCH_2_NC)AuCl], **1**. Complex **1** was also treated with potassium benzothiazole-2-thiolate (Kbt) and potassium benzoimidazole-2-thiolate (Kbi) in a 1:1 molar ratio and yielded complex (PhCH_2_NC)Au(κ^1^-S-bt)], **2a**, and (PhCH_2_NC)Au(κ^1^-S-bi)], **2b**, respectively, through a salt metathesis reaction (Scheme 1). Both complexes were air-stable, colorless solids which were obtained in a good yields and characterized using NMR and elemental analysis.

The ^1^H and ^13^C{^1^H} NMR spectra of **1**, **2a**, and **2b** (in CDCl_3_) displayed signal resonances due to the PhCH_2_NC ligand in expected regions (similar to the free ligand, with slight shifts) and a simple pattern for the thiolate ligands (50, 51). Furthermore, C^e^ and C^f^ of PhCH_2_NC ligand indicated a resolving coupling with nitrogen nucleus (^14^N) in the ^13^C{^1^H} NMR spectra of synthesized complexes which is characteristic feature for numerous isocyanide ligand and their complexes (15, 16 and 52). 

*Biological Activity studies*

The *in-vitro* cytotoxic activity of **2a** and **2b** were evaluated on three cancer cell lines including human ovarian (SKOV3), lung (A549), and breast (MCF-7) carcinoma. As shown in Table 1, **2a**, the exhibited comparable cytotoxicity on the investigated cell lines with cisplatin. It showed a good anti-proliferative activity with IC_50_ of 19.46, 11.76, and 13.27 μM compared with those measured for cisplatin (7.78, 13.27 μM and 11.69 μM, against A549, SKOV3 and MCF-7 cell lines, respectively). **2b** also showed generally a moderate antitumor activity especially against MCF-7 cell line with IC_50_ of 19.14 μM. Interestingly, **1** showed better antitumor activity against all the studied cancer cell lines compared to **2b**.

To investigate the selectivity between cancer and the normal cell line, the effects of the synthesized gold(I) complexes on the proliferation of the nontumoral cell line (MCF-10A; non-tumorigenic epithelial breast cell line) was also acquired. The results showed good selectivity between the tumorigenic and non-tumorigenic cell lines.

*Molecular docking analysis*

It was observed that gold acts as an anticancer agent through different mechanisms such as inhibition of the thioredoxinreductase (TrxR), gluthationereductase (GR) enzymes, and intercalation with the DNA (53). Hence, molecular docking simulation studies were conducted to determine the specific binding site and binding mode of the synthesized gold complexes to DNA and TrxR as their proposed targets.

The docking binding energies of the synthesized Au(I) complexes with DNA and TrxR targets are shown in Table 2. The lowest docking binding energies (kcal/mol) in Autodock dlg output file was considered as response in each run. As summarized in Table 2, **2a**, the best compound in the cytotoxic activity, showed also better energy regarding binding to TrxR active site compared to **2a**. These results suggest TrxR as the main target of these gold(I) anticancer agents. The ΔG_bind_ values of the best docked poses of these compounds are within the range of -7.71 to -13.86 kcal.mol^-1^ for DNA and -7.80 to -12.26 kcal.mol^-1^ for TrxR. The validity of the docking procedure was maintained by re-docking of Auronofin, the co-crystal ligand of TrxR, into 3D structure of TrxR. All the docking protocols were done on validated structures with RMSD values below 2 Å. 

**Figure 1:**
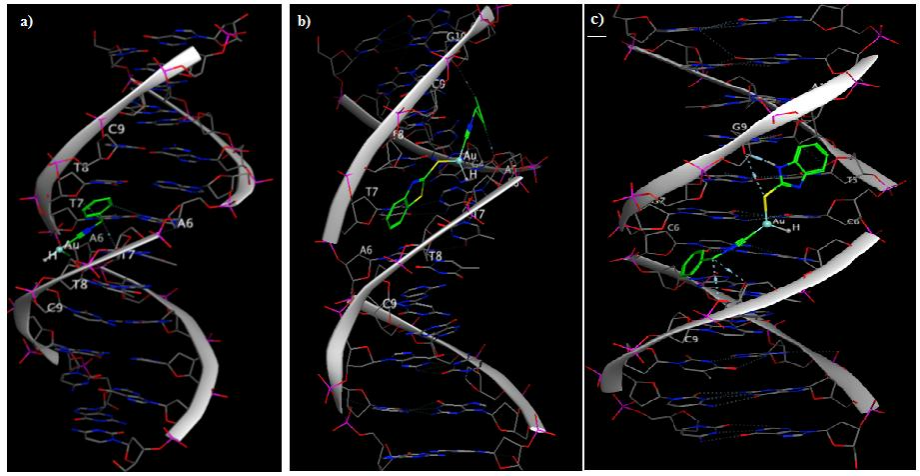
Molecular docking simulation studies of (a)** 1**, (b) **2a** with DNA (PDB ID: 1BNA), and (c) **2b** with DNA (PDB ID: 3CO3).

The docked model suggested that the compound **1** interacted with the minor groove of DNA with -7.71 kcal/mol binding energy through its chloro group with A6, and the benzyl CH_2_ group with T7 and T8 base pairs in the minor groove of DNA (PDB ID: 1BNA) (Figure 1a). The main interaction of **2a** was through hydrogen bonding of benzothiazole sulfur group with T7 in the minor groove of DNA (Figure 1b). **2b** interacted with the minor groove of DNA (PDB ID: 3CO3) with -8.55 Kcal/mol binding energy through its sulfur group attached to the gold atom with G9, benzothiazole nitrogen group with G9, gold atom with G7 and the benzyl CH_2_ group with T8 base pairs (Figure 1c).

**Figure 2:**
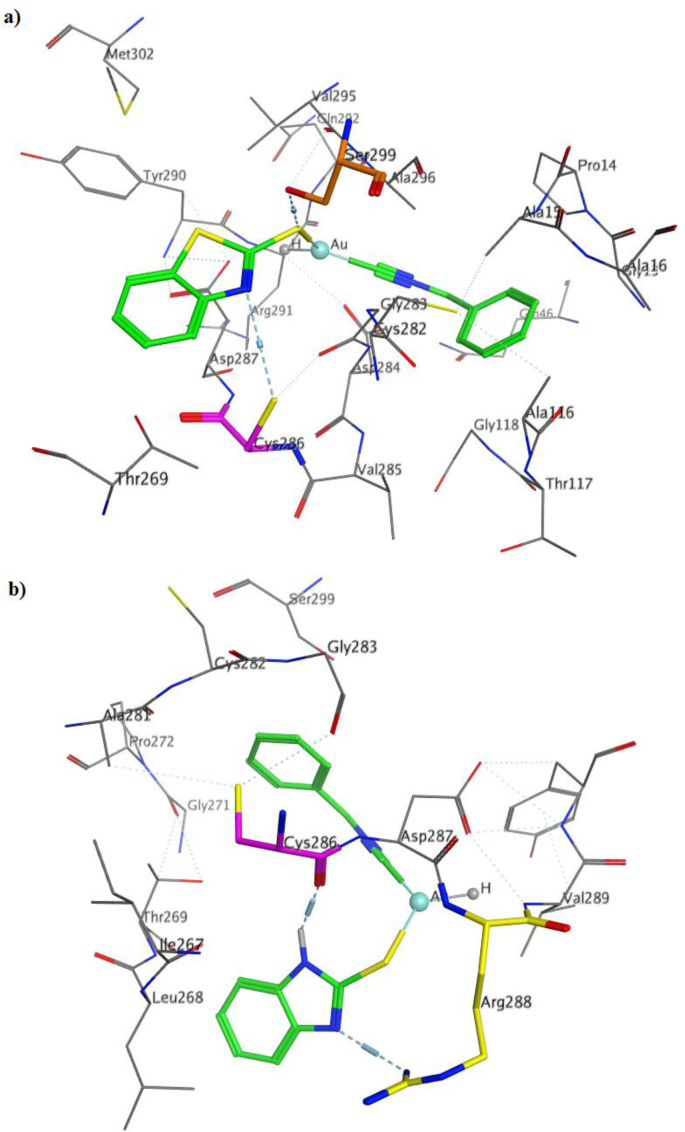
3D ligand-receptor interactions of (a)** 2a**, and (b) **2b** with TrxR (PDB code: 4CBQ).

The most important interactions of **2a** in binding to TrxR were the interaction of gold atom with Asp287, isocyande nitrogen group with Asp284, sulfur group attached to the gold atom with Ser299 and benzothiazole nitrogen group with Cys286 (Figure 2a). Binding mode of compound **2b** with TrxR showed that the nitrogen of isocyanide are involved in the acceptor hydrogen bonding with residue Asp287, and benzothiazole nitrogen groups with Cys286 and Arg288 (Figure 2b).

**Table 1:** *In-vitro* cytotoxic activity of gold complexes against cancerous and non-cancerous cell lines

**Name**	**IC**_50_ ** (μM ± SD)**			
	**A549**	**SKOV3**	**MCF-7**	**MCF-10A**
**1`** **2a**	25.18 ± 1.63 19.46 ± 1.15	28.73 ± 2.29 11.76 ± 1.49	15.67 ± 3.87 13.27 ± 3.37	58.35 ± 1.39 47.16 ± 1.28
**2b**	32.75 ± 1.47	22.52 ± 2.23	19.14 ± 1.28	45.08 ± 2.61
cisplatin	7.78 ± 0.54	13.27 ± 1.23	11.69 ± 1.57	28.42 ± 2.45

**Table 2:** Molecular docking studies of gold complexes on DNA and TrxR targets

	**Docking binding energy (kcal/mol)** ^a^				
Ligand/Receptor	1BNA^b^	1LU5^c^	3CO3^d^	198D^e^	4CBQ^f^
**1**	-7.71	-6.12	-6.57	-6.68	-7.80
**2a**	-11.76	-7.47	-8.24	-8.75	-12.26
**2b**	-13.86	-7.60	-8.55	-8.27	-11.69
Cisplatin	-	-4.44	-4.81	-4.71	-
Auronofin	-	-	-	-	-4.81

## Conclusion

In this study, two novel sulfur-based gold complexes are reported. Complex **1** is readily synthesized by replacement of dimethylsulfide ligand in **A** with benzyl isocyanide. In a salt metathesis reaction, an anion exchange between **1** and potassium benzothiazole-2-thiolate (Kbt) or potassium benzoimidazole-2-thiolate (Kbi), led to formation **2a** or **2b**, respectively. These complexes are fully characterized by NMR and elemental analysis. The cytotoxic activities of **2a** and **2b** against various cancer cell lines revealed that **2a** has reasonable IC_50_ with higher potency than **2b**. The evaluation of their cytotoxicity against the non-tumorigenic epithelial breast cell line (MCF-10A), showed good selectivity between the tumorigenic and non-tumorigenic cell lines. Complex **2a** showed a good anti-proliferative effect which is even higher than cisplatin against MCF-7 cell line.
